# Correlation Between Pathological and MRI Radiological Tumor Responses to Neoadjuvant Chemotherapy in Non‐Luminal Breast Cancer: A Single Institution Experience

**DOI:** 10.1002/cnr2.70275

**Published:** 2025-07-13

**Authors:** Wesal M. Eldehna, Fawzy Elbarbry, Abdul Hameed Hassan, Rafat Abu Shakra, Ahmed Elaryan, Ola Mousa Abdelfattah Elnady, Elshaimaa Mohamed Mohamed

**Affiliations:** ^1^ Department of Clinical Oncology and Nuclear Medicine Faculty of Medicine, Mansoura University Mansoura Egypt; ^2^ Department of Oncology International Medical Center Jeddah Saudi Arabia; ^3^ School of Pharmacy, Pacific University Hillsboro Oregon USA; ^4^ Department of Family Medicine International Medical Center Jeddah Saudi Arabia; ^5^ Department of Pathology and Laboratory Medicine International Medical Center Jeddah Saudi Arabia; ^6^ Department of Medical Imaging International Medical Center Jeddah Saudi Arabia; ^7^ Department of Diagnostic Radiology Faculty of Medicine, Zagazig University Zagazig Egypt

**Keywords:** breast cancer, correlation, magnetic resonance imaging, neoadjuvant chemotherapy, tumor response

## Abstract

**Background:**

Neoadjuvant chemotherapy (NACT) is the standard treatment for patients with locally advanced breast cancer. In recent years, it has also been used for early‐stage triple‐negative breast cancer (TNBC) and human epidermal growth factor receptor 2‐positive (HER2 ^+^) breast cancers.

**Objectives:**

Our hypothesis asserts a correlation between breast radiological and pathological response post‐NACT in non‐luminal breast cancer patients. We also aimed to determine the predictive value of MRI in predicting response in these patients. Radiologist agreement upon radiological MRI response is also highlighted in the current study.

**Methods:**

A retrospective study to evaluate MRI's accuracy for assessing tumor response to NACT in early and locally advanced non‐metastatic breast cancer patients in comparison with pathological assessments. We enrolled cases that were treated with neoadjuvant chemotherapy between December 2019 and November 2023.

**Results:**

Dynamic MRI's sensitivity to detect tumor response is 86.4%, its specificity is 96.8%, and its accuracy is 92.4%, with a significant *p*‐value. So, the high correlation between measurements of residual disease detected by MRI and those detected by pathological assessment supports the use of MRI for imaging assessment during NACT. The two radiologists involved in our study were in good agreement in their assessment of radiological response (Kappa: 0.801, sensitivity: 85.7%, specificity: 93.8%, and accuracy: 90.6%).

**Conclusions:**

The accuracy of imaging assessment by breast MRI during NACT is validated by the greater correlation between measurements of residual disease on MRI and pathology, establishing its role as the most precise imaging modality. We strongly advocate for the dependable role of MRI in monitoring breast lesions during neoadjuvant therapy, especially in non‐luminal breast cancer cases, regardless of whether they present as mass or non‐mass enhancement patterns.

## 1 Introduction

Breast cancer is a major global health issue, with around 2.3 million new cases reported in 2020, making it the most common cancer globally. This highlights the urgent need for ongoing research, early detection, and effective treatment options. As awareness and screening efforts improve, survival rates have also increased, emphasizing the importance of early intervention in combating this disease [1].

Treatment decisions for breast cancer are guided by factors such as the pathological type, tumor size, receptor status, molecular subtype, and cancer stage. Current therapeutic options include surgery and radiation for locoregional control, as well as chemotherapy and endocrine therapy for systemic management. Systemic treatments can be given in either the neoadjuvant (pre‐surgical) or adjuvant (post‐surgical) setting, with no significant differences seen in overall survival (OS) or disease‐free survival (DFS) [2].

Neoadjuvant chemotherapy is the standard treatment for patients with locally advanced breast cancer. More recently, it has also been utilized for early‐stage triple‐negative breast cancer (TNBC) and human epidermal growth factor receptor 2‐positive (HER2+) breast cancers [3]. These two aggressive tumor subtypes are more chemosensitive due to their high cellular proliferation, and therefore are preferred to be treated with NACT, even in an early stage [4]. The advantage of NACT is to decrease the size of preoperative tumors and to reduce the rate of tumor progression, raising the rate of success for breast‐conserving surgery, while reducing the mastectomy rate. Multiple studies have found a connection between the long‐term outlook for breast cancer and how patients respond to neoadjuvant chemotherapy; specifically, patients who achieve a pathological complete response (pCR) tend to have better DFS and OS [5].

The cancer's molecular subtype highly influences the response to NACT. Following completion of NACT, treatment decisions are guided by clinical and radiological evaluations of tumor response, utilizing various imaging modalities such as mammography, ultrasound (US), positron emission tomography (PET), and contrast‐enhanced magnetic resonance imaging (CE‐MRI) [6].

Mammography and breast US were traditionally the most commonly utilized imaging modalities for tumor diagnosis. However, they have shown limited accuracy in evaluating residual tumors after NACT, due to treatment‐induced fibrosis and tumor fragmentation, as well as their inability to assess the dynamic functional characteristics of the lesion [7].

Breast MRI is considered the most accurate imaging tool for assessing the response during and after neoadjuvant chemotherapy (NACT). Several studies have shown that breast MRI is superior to mammography and ultrasound in evaluating NACT efficacy. Recently, contrast‐enhanced mammography has been exploited in predicting the pathological response to NACT and to detect changes in the tumor's morphology and size [5]. Dynamic contrast‐enhanced MRI (DCE‐MRI) offers insights into the pathophysiological response of tumors to NACT, enabling earlier and more accurate evaluation of treatment response compared to anatomical imaging techniques. It detects tumor angiogenesis and related alterations in microcirculation and contrast uptake, due to the increased permeability of newly formed vessels within the growing tumor [7].

Several studies have shown that breast cancers with non‐mass enhancement (NME) on breast MRI often have lower agreement between MRI results and surgical pathology. Conversely, other research has emphasized the higher predictive accuracy of MRI in evaluating treatment response to NACT in specific subtypes.

Currently, there are no standardized criteria to identify imaging biomarkers that can reliably predict a patient's radiological response to NACT. Therefore, the current study aimed to assess the predictive value of MRI, particularly in TNBC and HER2+ subtypes, which may present as either mass or non‐mass enhancement on imaging.

Our research was conducted to demonstrate the MRI reliability for predicting pathologic complete response (pCR) and to emphasize its critical role in surgical planning for non‐luminal breast cancer. We focused on assessing the positive predictive value (PPV) and negative predictive value (NPV) of MRI for pCR following NACT. Additionally, we analyzed the extent of interobserver variability among breast radiologists interpreting MRI results, with the goal of establishing consensus imaging markers to more accurately predict treatment response.

## 2 Materials and Methods

### 2.1 Study Population

**FIGURE 1:**
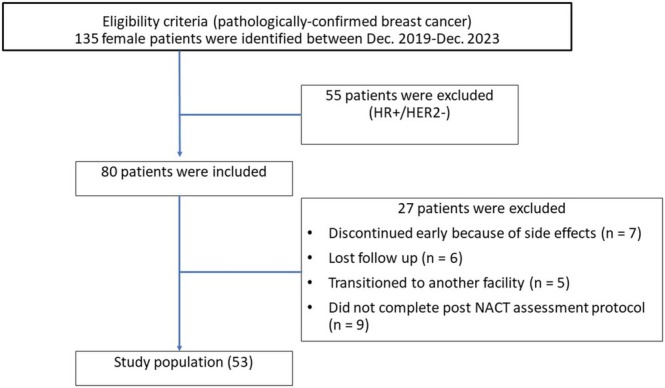
Flow chart for the study population.

The research received formal approval from the Institutional Review Board, under the assigned protocol number 2023–11‐234, of the International Medical Center (IMC) in Jeddah, Saudi Arabia. They waived the need for written informed consent. Our study reviewed retrospectively 135 female patients with invasive breast cancer who received NACT and had their condition assessed by breast MRI at IMC between December 2019 and November 2023. To be included, patients had to be 18 years or older, have pathologically confirmed invasive ductal carcinoma that was either triple‐negative or HER2+ subtype, have measurable disease according to the Response Evaluation Criteria in Solid Tumors, version 1.1, and have a performance score of 2 or less. Exclusion criteria included previous chemotherapy, double malignancy, and lost follow‐up. Baseline patient's demographics (such as age, sex, and menopausal status), histopathological subtypes, nodal involvement, chemotherapy protocol given, and image findings by MRI (pre‐ and post‐chemotherapy) were retrospectively collected from the electronic medical records system. The NACT regimen was determined according to the pathological subtype and the treating physician's choice, as per treatment guidelines. Upon applying the inclusion and exclusion criteria, 55 patients were excluded due to their receptor status (HR+/HER2−), as we aimed to conduct our research on non‐luminal breast cancer only. 18 patients discontinued early due to side effects (= 7), lost follow‐up (= 6), and transition to another facility (= 5), and 9 patients were excluded due to unavailable imaging work‐up post‐NACT. Therefore, 53 patients were able to be included in the study, as shown in Figure 1.

The consent was signed by the patient after explaining the treatment's side effects. CE‐MRI was performed after finishing the neoadjuvant course to evaluate treatment response radiologically before the surgical excision. A pathological reassessment of the post‐surgery specimen was performed to state the treatment response and to compare it with the radiological response.

### 2.2 Radiological Tumor Response Assessment via MRI

All participants in this study underwent two dynamic MRI scans: one prior to and another 2 weeks after ending the neoadjuvant chemotherapy (NACT) course. Imaging was conducted using a 1.5‐Tesla MRI scanner (Magnetom Avanto Siemens, USA) equipped with a dedicated breast coil. The protocol included a dynamic contrast‐enhanced sequence utilizing a bilateral, three‐dimensional, fat‐suppressed, T1‐weighted gradient‐echo technique. The imaging parameters were as follows: repetition time (TR) between 4 and 10 ms, minimum echo time (TE), flip angles from 10° to 20°, a field of view ranging from 26 to 36 cm, frequency (read) matrix between 384 and 512, phase encoding matrix of at least 256, and an in‐plane spatial resolution of 1.4 × 1.4 mm. Section thickness did not exceed 2.5 mm, with temporal resolution between 80 and 100 s, using axial orientation in the prone position.

A standardized protocol for administering contrast agent was followed, delivering it at 2 mL/s, immediately followed by a 20 mL saline flush. DCE‐MRI scans were obtained before and at several time points after contrast administration (Gadoterate, 0.1 mmol/kg body weight, injected at 1.5 mL/s, followed by a 10 mL saline flush at the same rate). The first post‐contrast sequence was captured 90 s after injection, with the final sequence at 6 min post‐injection.

Additional sequences included axial fat‐suppressed T2‐weighted imaging (T2WI), diffusion‐weighted imaging (DWI) with *b*‐values of 0 and 1000 s/m^2^, and DCE fat‐suppressed T1‐weighted sequences covering both early and late enhancement phases were acquired separately with an IV injection of Gadolinium‐DTPA (0.2 mL/kg). The first post‐contrast images were collected 60 s later when gadolinium‐DTPA was injected and then six subsequent scans were obtained.

All MRI examinations were retrospectively analyzed by two board‐certified radiologists, EM and AE, who have 25 and 20 years of specialized experience in breast imaging, respectively. To ensure unbiased interpretation, both radiologists were blinded to the corresponding pathological findings. Image assessments were conducted in accordance with the criteria established by the American College of Radiology's Breast Imaging Reporting and Data System (ACR BI‐RADS). For each lesion, they assessed characteristics such as size, shape (oval, round, or irregular), margin (circumscribed, spiculated, or irregular), and internal enhancement pattern (homogeneous, rim‐enhancing, or heterogeneous). The smallest diameter of any visible lymph nodes was also measured.

Tumor response was classified using the Response Evaluation Criteria in Solid Tumors (RECIST) version 1.1. A complete response (CR) was defined as the total disappearance of index lesions, while a partial response (PR) was defined as a reduction of at least 30% in the sum of the diameters of target lesions.

### 2.3 Pathological Tumor Response Assessment

The initial diagnosis of breast cancer was established using core needle biopsies performed under ultrasound guidance in the radiology department. All biopsy specimens were fixed in 10% neutral buffered formalin for 6 to 48 h, with cold ischemia times kept under 1 h. Standard procedures for tissue processing, embedding, sectioning, and hematoxylin and eosin (H&E) staining were carried out in the pathology department. Histological examination confirmed the diagnosis, and immunohistochemical staining for estrogen receptor, progesterone receptor, and HER2 was performed using antibody clones SP1, IE2, and 4b5, respectively (UltraView, Universal DAB Detection Kit, VENTANA BenchMark XT/LT).

Post‐neoadjuvant surgical specimens were re‐evaluated pathologically. Lumpectomy or mastectomy specimens were fixed in 10% neutral buffered formalin for 24 to 72 h, with cold ischemia time maintained below 1 h. The specimens were grossly described, margins inked, and thinly sliced (3–5 mm), followed by overnight fixation. The tumor bed was identified visually and by delicate palpation, with extensive sampling of the tumor bed and any firm or suspicious tissue from the surgical margins, as well as nipple and/or skin if present. Sentinel and non‐sentinel lymph nodes were also sampled after thin slicing (2 mm). Histopathological examination was conducted to determine the presence or absence of pathological complete response (pCR), defined as the absence of invasive cancer in both the breast and axillary lymph nodes following completion of NACT.

### 2.4 Statistical Analysis

Sample size was determined using Stata Statistical Software: Release 17 (StataCorp LLC, College Station, TX, 2021). The reported prevalence of breast cancer in Africa is 6.2%, with approximately 55% of cases being non‐luminal breast cancer [8], and the prevalence of non‐luminal breast cancer estimated at 3.41% (6.2% × 55%) [9]. Using a 95% confidence level, 5% confidence limits, and an anticipated frequency of 3.41%, the minimum required sample size was calculated to be 51 non‐luminal breast cancer cases. To accommodate potential attrition and enhance statistical power, the final sample size was increased to 53 cases.

Data analysis was performed using IBM SPSS Statistics for Windows, Version 25.0 (IBM Corp., Armonk, NY, 2017). The Chi‐square test was used to evaluate associations between qualitative variables, while Fisher's exact test was applied when expected counts were < 5 in more than 20% of cells. The kappa statistic was calculated to assess agreement between methods. The level of agreement was evaluated based on the interpretive framework proposed by Altman, which categorizes the strength of concordance as follows: values below 0.20 indicate “poor” agreement, scores ranging from 0.21 to 0.40 reflect a “fair” level of consistency, values between 0.41 and 0.60 denote “moderate” agreement, the 0.61 to 0.80 range corresponds to a “good” level of reliability, and scores from 0.81 to 0.99 signify “very good” or “almost perfect” agreement.

Performance metrics were calculated as follows:

*Sensitivity*: Probability of a positive test when the reference method is positive = (true positives)/(true positives + false negatives).

*Specificity*: Probability of a negative test when the reference method is negative = (true negatives)/(true negatives + false positives).

*Positive Predictive* Value (*PPV*): Probability of a positive result by both methods when either is positive = (true positives)/(true positives + false positives).

*Negative Predictive* Value (*NPV*): Probability of a negative result by both methods when either is negative = (true negatives)/(false negatives + true negatives).

*Positive Likelihood Ratio* (*PLR*): Probability of a positive result in patients with the disease divided by that in patients without the disease = (sensitivity)/(1−specificity).

*Negative Likelihood Ratio* (*NLR*): Probability of a negative result in patients with the disease divided by that in patients without the disease = (1−sensitivity)/(specificity).

Continuous variables were analyzed using Student's *t*‐test. To determine potential risk factors, logistic regression analysis was employed as the primary statistical method. Associations were deemed statistically significant when the *p*‐value was below the threshold of 0.05, corresponding to a 95% confidence level, indicating a < 5% probability that the observed results occurred by chance.

## 3 Results

According to Table 1, the mean age of the 53 breast cancer patients was 50.2 years, with a standard deviation of 10.9 years. Among these patients, more than half (58.5%) were not yet menopausal. TNBC (54.7%) was the most prevalent cancer subtype, followed by HER2 + (34%) and TPBC (11.3%). There were 64.2% positive biopsies of lymph nodes. Physicians assessed patient response in a variety of ways, of which 37.7% of patients showed partial response by radiological evaluation and 41.5% by pathological assessment. Of these patients, 37.7% had positive post‐NACT MR index mass size, index mass kinetics, and radiologically suspicious lymph nodes. Also, this table shows the assessment of the radiological response by two expert radiologists, where radiologist 1 classified 60.4% of patients as CR and 39.6% as PR, while radiologist 2 classified 62.3% of patients as CR and 37.7% as PR.

**TABLE 1:** Baseline clinicopathological factors of study population.

Characteristics	All cohort (*n* = 53)
Age (years)	
Mean ± SD	50.2 ± 10.9
Median (Minimum—Maximum)	51 (32%–78%)
Menopause	
Premenopausal	31 (58.5%)
Postmenopausal	22 (41.5%)
Subtype	
TPBC	6 (11.3%)
TNBC	29 (54.7%)
HER2	18 (34.0%)
Biopsied lymph nodes	34 (64.2%)
Radiological response	
CR	33 (62.3%)
PR	20 (37.7%)
Pathologic response	
CR	31 (58.5%)
PR	22 (41.5%)
Post NACT MR index mass/non‐mass measurements	20 (37.7%)
Index mass/non‐mass kinetics	
Radiologically suspicious lymph nodes	20 (37.7%)
Radiological response	
*Radiologist #1*	
CR	32 (60.4%)
PR	21 (39.6%)
*Radiologist #2*	
CR	33 (62.3%)
PR	20 (37.7%)

*Note:* Non‐numerical data was expressed by using no. (%).

Abbreviations: CR, complete response; PR, partial response; SD, standard deviation.

Table 2 shows a statistically significant association between post‐NACT MRI findings and different parameters (*p* < 0.05). Regarding breast cancer subtypes, a significant majority (85%) of patients with a remaining mass had TNBC, versus 36.4% of patients without a remaining mass. Only 10% of patients with a remaining mass had HER2, versus 48.5% of patients without a remaining mass. On the other hand, there was no statistically significant difference regarding TPBC type. Radiologic and pathologic findings indicate 100% and 95% of patients with a remaining mass/non‐mass showed partial responses, respectively, compared to 0% and 9.1% of patients without a remaining mass/non‐mass. Also, all patients with a remaining mass/non‐mass had abnormal index mass/non‐mass kinetics and positive suspicious lymph nodes, and all patients without a remaining mass/non‐mass had no index mass/non‐mass kinetics or suspicious lymph nodes. However, there was no statistically significant association between post‐NACT MRI findings and menopause or biopsied LN (*p* ≥ 0.05).

**TABLE 2:** Factors associated with post‐NACT MRI findings.

	Post‐NACT MR index mass/non‐mass measurements		*p*
	No (*n* = 33)	Yes (*n* = 20)	
Menopause			
Premenopausal	19 (57.6%)	12 (60%)	0.862
Postmenopausal	14 (42.4%)	8 (40%)	
Subtype			
TPBC	5 (15.2%)	1 (5%)	0.258
TNBC	12 (36.4%)	17 (85%)	0.001*
HER2	16 (48.5%)	2 (10%)	0.004*
Biopsied LN			
Negative	14 (42.4%)	5 (25%)	0.200
Positive	19 (57.6%)	15 (75%)	
Radiologic response			
CR	33 (100%)	0 (0%)	< 0.001*
PR	0 (0%)	20 (100%)	
Pathologic response			
CR	30 (90.9%)	1 (5%)	< 0.001*
PR	3 (9.1%)	19 (95%)	
Index mass/non mass kinetics			
No	33 (100%)	0 (0%)	< 0.001*
Yes	0 (0%)	20 (100%)	
Radiologically‐Suspicious LN			
Negative	33 (100%)	0 (0%)	< 0.001*
Positive	0 (0%)	20 (100%)	

*Note:* * indicates significant difference.

To assess the degree of agreement among the 2 radiologists, Kappa test was performed. As Table 3 shows, there was a good agreement between the two radiologists in their assessment of response (Kappa: 0.801, sensitivity: 85.71%, specificity: 93.75%, and accuracy: 90.57%).

**TABLE 3:** Agreement between radiologist 1 and radiologist 2.

		Radiologist 1										Accuracy
		CR (32)	PR (21)	*p*	Kappa	Sensitivity	Specificity	PPV	NPV	PLR	NLR	
Radiologist 2	CR	30 (93.8%)	3 (14.3%)	< 0.001	0.801	85.7%	93.7%	90.0%	90.9%	13.7%	0.15%	90.6%
	PR	2 (6.30%)	18 (85.7%)									

*Note:* Non‐numerical data was expressed by using no. (%).

Abbreviations: NLR, negative likelihood ratio; NPV, negative predictive value; PLR, positive likelihood ratio; PPV, positive predictive value.

In our study, any discrepancies between radiologists regarding the classification of post‐NACT MRI responses were resolved through consensus review. The results shown in Table 4 illustrate a very good agreement between pathologic response and each of the radiologic responses regarding MR index mass/non‐mass measurements on post‐NACT MRI (Kappa: 0.842, Sensitivity: 86.4%, Specificity: 96.8%, and Accuracy: 92.4%) with a *p* value < 0.05. On the other hand, a poor agreement and non‐statistically significant difference were found between biopsied LN and radiologically suspicious results, as depicted in Table 5.

**TABLE 4:** Diagnostic performance and correlation between radiologic and pathologic response of breast mass/non‐mass pattern.

	Pathologic response											
		CR (31)	PR (22)	*p*	Kappa	Sensitivity	Specificity	PPV	NPV	PLR	NLR	Accuracy
Radiologic response	CR	30 (96.8%)	3 (13.6%)	< 0.001	0.842	86.4%	96.8%	95.0%	90.9%	26.8	0.14	92.4%
	PR	1 (3.2%)	19 (86.4%)									

*Note:* Non‐numerical data was expressed by using no. (%).

Abbreviations: NLR, negative likelihood ratio; NPV, negative predictive value; PLR, positive likelihood ratio; PPV, positive predictive value.

**TABLE 5:** Diagnostic performance and correlation between radiologic and pathologic response of axillar lymph nodes (LN).

	Biopsied LN											
		Negative (19)	Positive (34)	*p*	Kappa	Sensitivity	Specificity	PPV	NPV	PLR	NLR	Accuracy
Radiologically Suspicious LN
	Negative	12 (73.7%)	19 (55.9%)	0.200	0.153	44.1%	73.7%	75.0%	42.4%	1.68	0.76	54.7%
Positive	5 (26.3%)	15 (44.1%)										

*Note:* Non‐numerical data was expressed by using no. (%).

Abbreviations: NLR, negative likelihood ratio; NPV, negative predictive value; PLR, positive likelihood ratio; PPV, positive predictive value.

Based on Table 6, the results showed that 85% and 77.3% of patients who had partial responses in radiologic and pathologic assessment, respectively, had TNBC. On the other hand, only 10% and 18.2% of patients who had partial radiologic and pathologic responses were HER2+, and this difference was statistically significant (*p* < 0.05). Also, there was a statistically significant association between partial response (PR) on radiology or pathology with positive index mass kinetics, radiologically suspicious lymph nodes, and change in size on post‐NACT MRI (*p* < 0.05). All patients with a PR on radiologic assessment and 86.4% of those with a PR on pathological evaluation exhibited positive index mass kinetics on imaging, radiologically suspicious lymph nodes, and a change in post‐NACT MRI index mass size. In contrast, none of the patients with a complete radiologic response (CR) and only 3.2% of those with a complete pathological response showed these features. However, there was no association between radiology or pathology responses and menopause (*p* ≥ 0.05). Logistic regression analysis was also performed, which reinforced the obtained results.

**TABLE 6:** Relation between radiologic response and pathologic response with different parameters.

	Radiologic response		*p*	OR (95% CI)	Pathologic response		*p*	OR (95% CI)
	CR (*n* = 33)	PR (*n* = 20)			CR (*n* = 31)	PR (*n* = 22)		
Menopause								
Premenopausal	19 (57.6%)	12 (60%)		Reference	19 (61.3%)	12 (54.5%)		Reference
Postmenopausal	14 (42.4%)	8 (40%)	0.862	0.94 (0.47–1.89)	12 (38.7%)	10 (45.5%)	0.623	1.19 (0.59–2.37)
Subtype								
TPBC	5 (15.2%)	1 (5%)	0.390	0.48 (0.14–1.67)	5 (16.1%)	1 (4.5%)	0.382	0.43 (0.12–1.51)
TNBC	12 (36.4%)	17 (85%)	0.001*	3.9 (1.78–8.66)	12 (38.7%)	17 (77.3%)	0.005*	2.80 (1.35–5.81)
HER2	16 (48.5%)	2 (10%)	0.004*	0.28 (0.12–0.68)	14 (45.2%)	4 (18.2%)	0.041*	0.45 (0.21–0.97)
Biopsied LN								
Negative	14 (42.4%)	5 (25%)		Reference	15 (48.4%)	4 (18.2%)		Reference
Positive	19 (57.6%)	15 (75%)	0.200	1.62 (0.78–3.40)	16 (51.6%)	18 (81.8%)	0.024*	2.41 (1.12–5.16)
Index mass kinetics								
No	33 (100%)	0 (0%)	< 0.001*	—	30 (96.8%)	3 (13.6%)	< 0.001*	Reference
Yes	0 (0%)	20 (100%)		—	1 (3.2%)	19 (86.4%)		19.7 (6.5–59.4)
Suspicious radiologically LN								
Negative	33 (100%)	0 (0%)	< 0.001*	—	30 (96.8%)	3 (13.6%)	< 0.001*	Reference
Positive	0 (0%)	20 (100%)		—	1 (3.2%)	19 (86.4%)		19.7 (6.57–59.4)
Post NACT MR index mass size								
No	33 (100%)	0 (0%)	< 0.001*	—	30 (96.8%)	3 (13.6%)	< 0.001*	Reference
Yes	0 (0%)	20 (100%)		—	1 (3.2%)	19 (86.4%)		19.7 (6.53–59.4)

*Note:* * indicates significant difference.

Abbreviations: CI, confidence interval; OR, odds ratio.

Based on Table 7, the results showed perfect agreement between pathologic and radiologic responses regrading patients with premenopausal and TNBC type of breast cancer (Kappa: 1, sensitivity: 100%, specificity: 100%, and accuracy: 100%). Also, there was a moderate agreement between pathologic and radiologic responses regarding postmenopausal patients (Kappa: 0.627, sensitivity: 87.5%, specificity: 78.6%, and accuracy: 81.8%) and patients who had HER2 + type of breast cancer (Kappa: 0.609, sensitivity: 100%, specificity: 87.5%, and accuracy: 88.9%).

**TABLE 7:** Association between radiologic response and clinicopathological factors.

	Radiologic response		Pathological response										
	CR (*n* = 33)	PR (*n* = 20)	CR (*n* = 31)	PR (*n* = 22)	*p*	Kappa	Sensitivity (%)	Specificity (%)	PPV (%)	NPV (%)	PLR	NLR	Accuracy (%)
Menopause													
Premenopausal	19 (57.6%)	12 (60%)	19 (61.3%)	12 (54.5%)	< 0.001	1	100	100	100	100	—	0	100
Postmenopausal	14 (42.4%)	8 (40%)	12 (38.7%)	10 (45.5%)	0.003	0.627	87.5	78.6	70.0	91.7	4.09	0.16	81.8
Subtype													
TPBC	5 (15.2%)	1 (5%)	5 (16.1%)	1 (4.5%)	0.624	0	0	80.0	0	80.0	0	1.25	66.7
TNBC	12 (36.4%)	17 (85%)	12 (38.7%)	17 (77.3%)	< 0.001	1	100	100	100	100	—	0	100
HER2	16 (48.5%)	2 (10%)	14 (45.2%)	4 (18.2%)	0.005	0.609	100	87.5	50.0	100	8	0	88.9

*Note:* Non‐numerical data was expressed by using no. (%).

Abbreviations: NLR, negative likelihood ratio; NPV, negative predictive value; PLR, positive likelihood ratio; PPV, positive predictive value.

**FIGURE 2:**
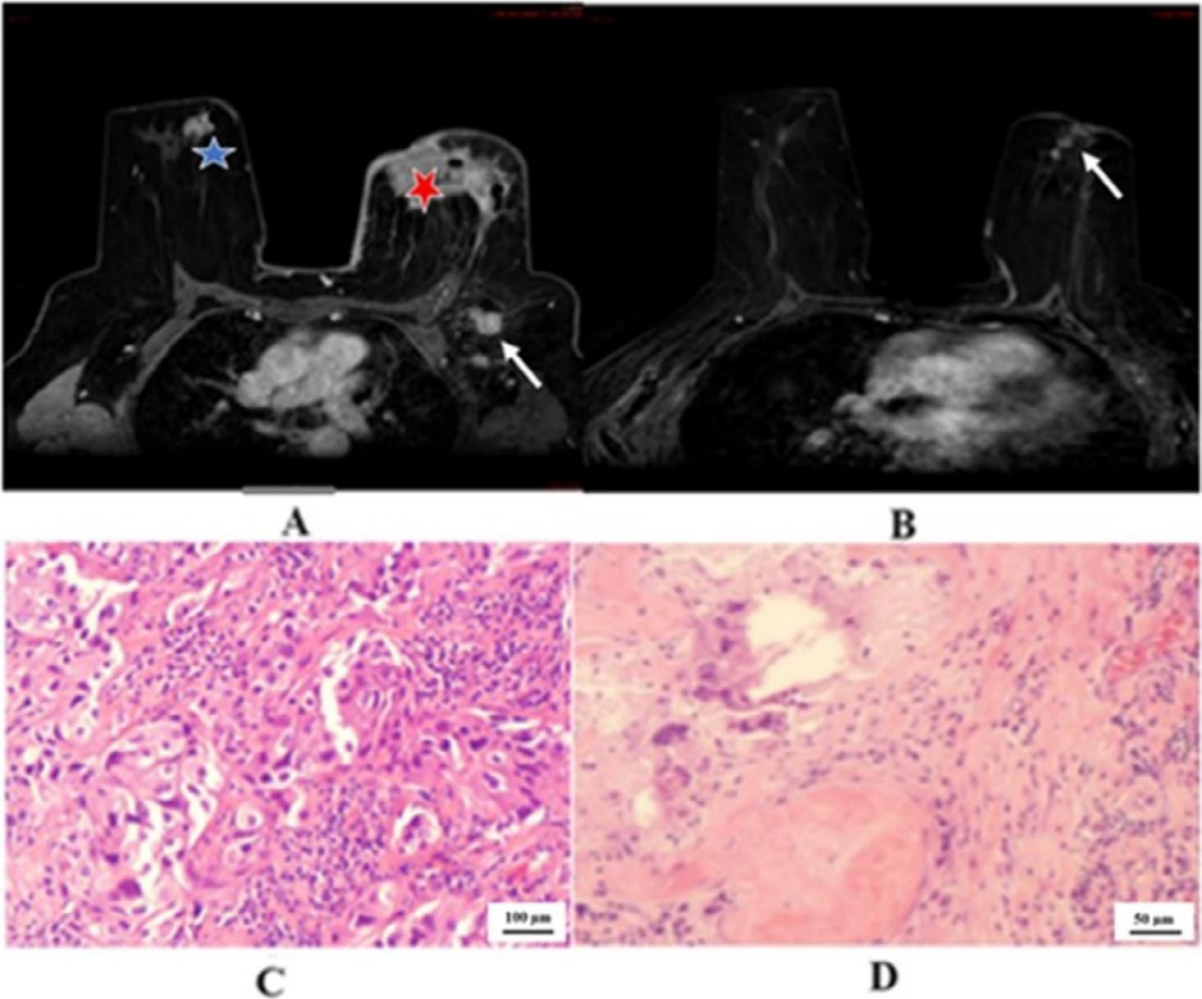
(A); pre‐NACT, DCE‐MRI T1 shows right breast spiculated retro areolar lesion (blue star) without nipple invasion. A large left malignant lesion (red star) with a biopsy clip inside invaded the nipple and skin and axillary suspicious LN (arrow). (B); Post NACT, DCE‐MRI T1 shows near total resolution of the previous lesion; however, residual linear enhancement is identified (radiological PR). The left breast shows an interval complete resolution with a residual multiple linear non‐mass‐like enhancement (radiological PR). (C); Core biopsy: Invasive ductal carcinoma, G 3. The tumor cells show large nuclei and prominent nucleoli (10X magnification, H&E stain). (D); Mastectomy specimen: The tumor bed shows fibrosis, histiocytic infiltrate, and chronic inflammation. No residual malignancy is identified (20X magnification, H&E stain), pathological CR.

**FIGURE 3:**
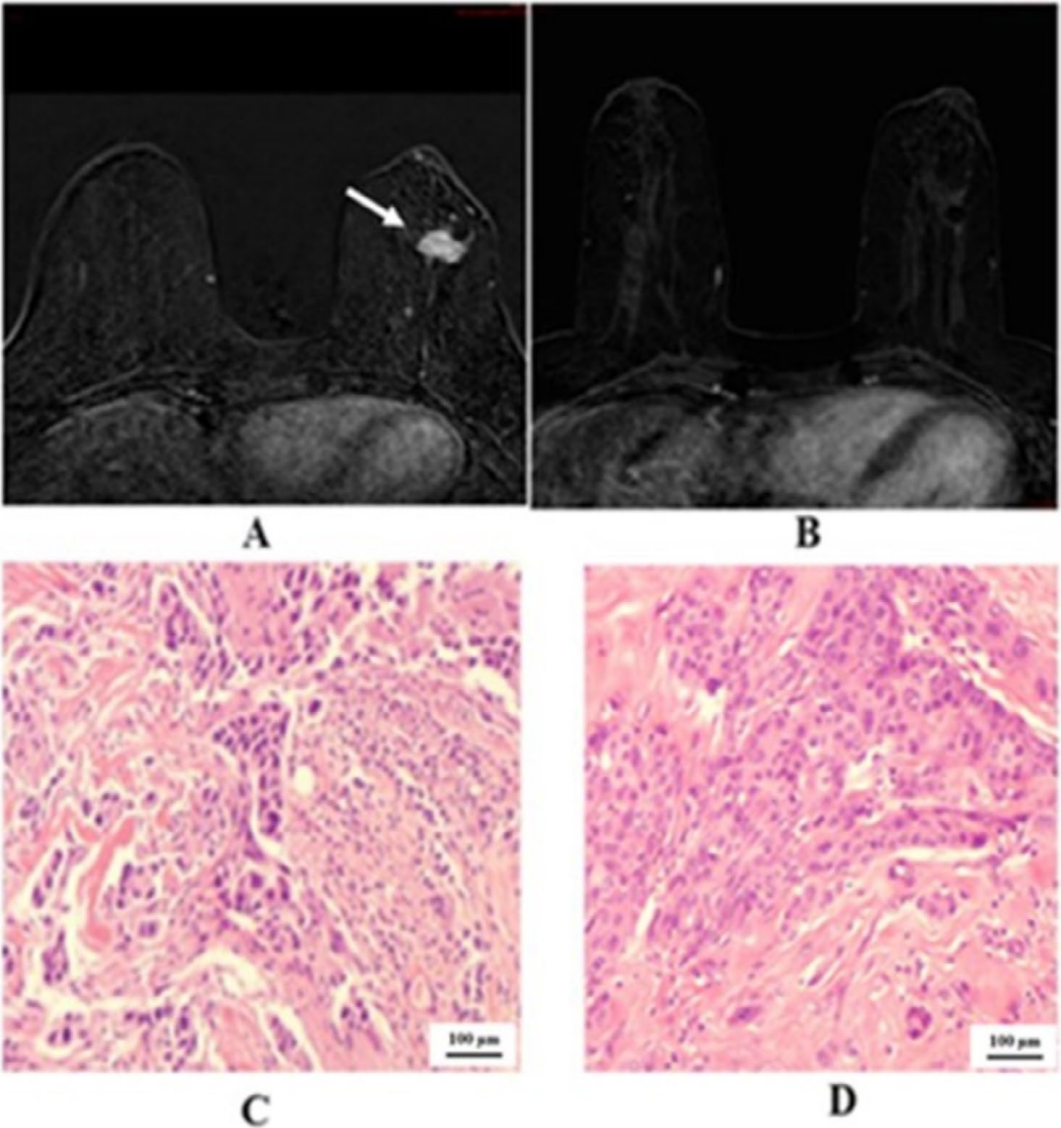
(A); pre‐NACT, DCE‐MRI T1 shows left breast spiculated mass (arrow). (B); post‐NACT, DCE‐MRI T1 shows complete resolution of the previous lesion in keeping with the radiological CR. (C); core biopsy showing invasive ductal carcinoma, grade 3. The tumor cells are arranged in trabeculae and solid sheets (10X magnification, H&E stain). (D); lumpectomy specimen. Small residual carcinoma is present. The tumor cells show large nuclei and prominent nucleoli (10X magnification, H&E stain), pathological PR.

**FIGURE 4:**
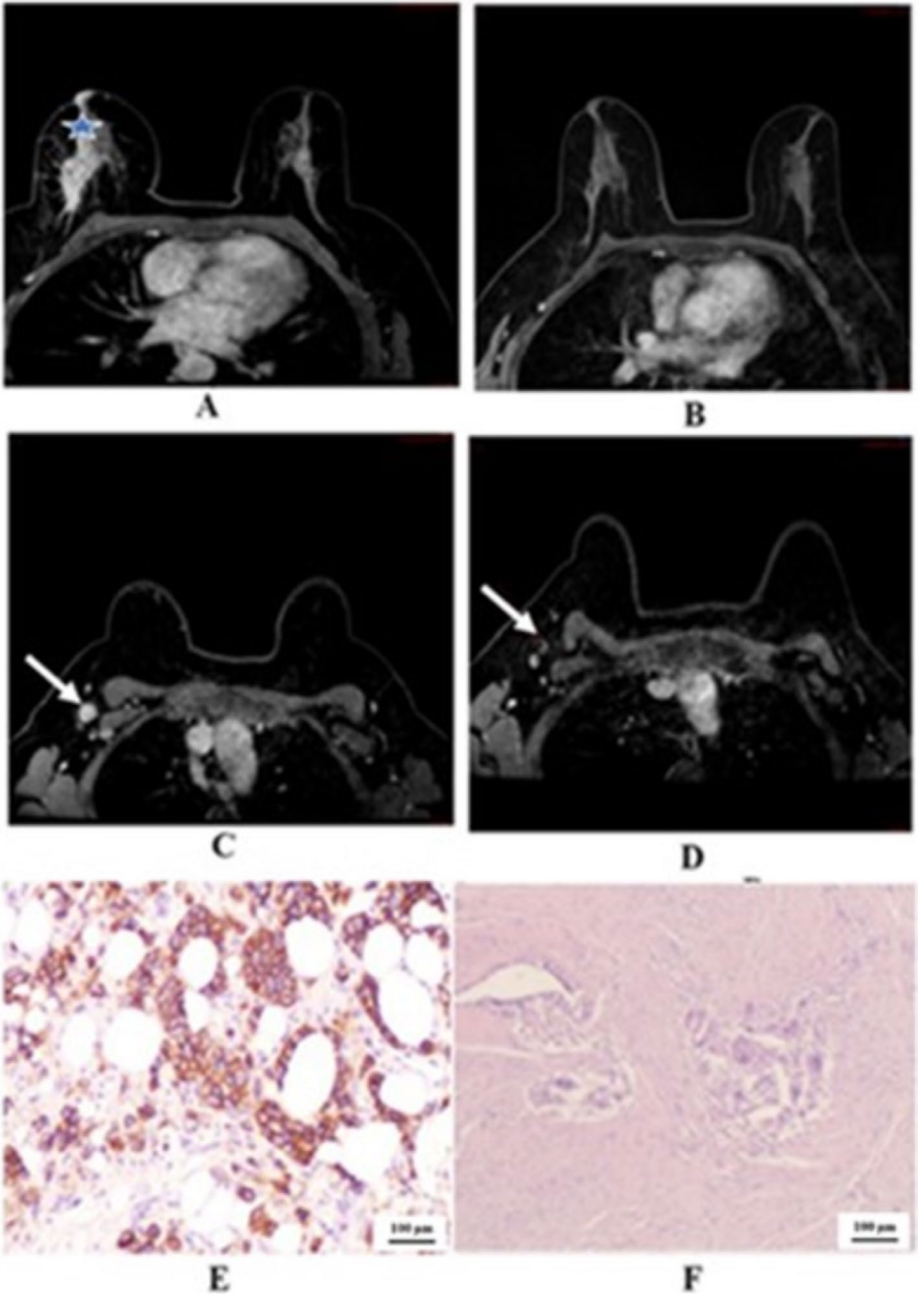
(A) and (C); pre‐NACT DCE‐MRI T1 shows a large segmental area of right non‐mass‐like heterogeneous enhancement extending toward the nipple with enhancement of the nipple (blue star). Associated multiple focal enhancing small masses corresponding to multicentric disease. Pathological lymph nodes with a biopsy clip inside (arrow). (B) and (D); Post NACT, DCE‐MRI T1 shows complete resolution. The axillary biopsied LN (arrow) retained the benign pattern in keeping with the radiological CR. (E); HER2: Immunostaining for HER‐2 is positive (score 3+) (10X magnification). (F); Mastectomy specimen. The tumor bed shows fibrosis, histiocytic infiltrate, and multinucleated giant cells. No residual malignancy is identified (10X magnification, H&E stain) in keeping with pathological CR.

We demonstrated the two cases in the study that showed negative predictive value. One case of right breast cancer with GIII invasive ductal carcinoma, positive hormonal receptor and HER2 overexpressing, in Figure 2 that showed pCR, but radiological PR. The other case of left breast cancer with GIII invasive ductal carcinoma, positive hormonal receptor and HER2 overexpressing, in Figure 3 that showed pPR, but radiological CR. In Figure 4 we illustrated a case of right breast cancer with negative hormonal receptor and HER2 overexpressing that showed pCR, and radiological CR with positive predictive value.

## 4 Discussion

For patients who have surgery after NACT, the standard definition of pCR is having no remaining invasive tumor in either the breast or the axillary lymph nodes [10]. In the current study, the pCR rate was 58.5%, consistent with the findings of Reig et al., who noted that because of the heterogeneous nature of breast cancer, pCR rates after NACT can vary widely from 0.3% to 50.3% depending on the tumor subtype [2].

According to the RECIST 1.1 criteria, MRI is the recommended imaging modality in the neoadjuvant setting for monitoring breast lesions [11]. The findings of this observational comparative effectiveness study reveal a statistically significant correlation between pathological response and radiologic assessments, with a Kappa coefficient of 0.84, a sensitivity of 86.4%, a specificity of 96.8%, and an overall accuracy of 92.5% (*p* < 0.05). These results align closely with the findings reported by Moo TA et al., who found MRI sensitivity for detecting residual disease to be between 63% and 88%, and specificity to range from 54% to 91% [12]. However, our findings differ from those of Kwak et al. [13] who reported that post‐NACT MRI predicted pathological response with an overall accuracy of 69%.

This discrepancy may be explained by differences in study design, specifically, our exclusion of luminal subtypes. Luminal tumors are more likely to present as non‐mass lesions, which often result in discordance between radiological and pathological assessments. In contrast, we focused on non‐luminal subtypes, which tend to be higher grade, exhibit greater proliferative activity, and are generally more responsive to NACT. Moreover, these subtypes predominantly present as mass lesions, which are more reliably assessed using imaging techniques.

Our findings align with those of Mukhtar et al. [14], who reported that well‐circumscribed mass lesions exhibit greater concordance between MRI findings and surgical pathology. Specifically, radiological CR in solid tumor phenotypes, particularly in hormone receptor‐negative cancers, was a strong predictor of pCR at surgery. In contrast, MRI demonstrated lower accuracy in predicting pCR for tumors that presented as non‐mass‐like or showed diffuse enhancement, where discrepancies between post‐NACT radiologic and pathologic evaluations were more pronounced.

Our results showed that all patients with a PR on imaging, and 86.4% of those with PR on pathology, exhibited positive kinetic features of the index mass or non‐mass enhancement, radiologically suspicious lymph nodes, and a measurable change in the size of the index lesion on post‐NACT MRI. In contrast, none of the patients with a CR on imaging, and only 3.2% with a CR on pathology, displayed these imaging characteristics.

Similar studies have noted that some participants exhibited partial radiological responses characterized by persistent non‐mass enhancement on dynamic MRI; however, subsequent pathological evaluation after surgery classified these cases as complete pathological responses [15, 16, 17]. Fibrosis induced by NACT can mimic residual tumor on dynamic MRI, leading to misclassification in some cases. As a result, approximately 13%–17% of patients without residual tumor on pathological assessment may still be classified as having achieved a pCR radiologically. Choi WJ et al. reported that non‐mass lesions are more prone to false‐negative MRI findings, noting three cases where dynamic MRI incorrectly indicated a complete radiological response [18].

Fukada et al. reported that tumors demonstrate diverse shrinkage patterns in response to NACT, including complete response, concentric shrinkage, and non‐concentric (or “crumbling”) shrinkage [19]. Distinguishing whether post‐treatment enhancement reflects carcinoma in situ, invasive cancer, or therapy‐related changes can be challenging, particularly in luminal subtypes presenting as non‐mass enhancement [20]. Tumor subtype has a significant impact on the rates of false‐positive and false‐negative interpretations on MRI. TNBC and HER2+ subtypes demonstrate the highest MRI accuracy in predicting pCR [19].

In our study, 85% of patients with partial radiologic responses and 77.3% of those with partial pathological responses were diagnosed with TNBC. In contrast, only 10% and 18.2% of patients with partial radiologic and pathological responses, respectively, had tumors that were HER2^+^. This difference was statistically significant (*p* < 0.05). These results are consistent with a previous study by Kwak et al. [13] which demonstrated that the correlation between residual disease on MRI and pathology varied significantly across molecular subtypes. The highest positive predictive value (PPV) was observed in luminal HR+/HER2‐ tumors (84%), while the lowest was seen in HER2+ tumors (57%). In some cases, post‐NACT MRI enhancement may be misinterpreted as residual disease. Further research is needed to examine the relationship between tumor grade, proliferation index, and imaging appearance after NACT. Our findings showed that radiological assessment of residual disease on dynamic contrast‐enhanced MRI by two expert radiologists yielded good interobserver agreement (κ = 0.80), with a sensitivity of 85.7%, specificity of 93.7%, and an overall accuracy of 90.6%. These results were in line with those of Lee et al. [21], who reported moderate inter‐reader agreement for intra‐tumoral T2 hyperintensity and peritumoral edema on ADC measurements, with kappa values of 0.529 and 0.539, respectively.

The notable inconsistency among radiologists in identifying pathological lymph nodes underscores a key limitation of current radiological assessment. MRI, in particular, has demonstrated a high false‐negative rate in evaluating the axilla post‐NACT, revealing its limitations in this context. This highlights the value of using radiological tools as a complementary approach rather than a substitute for pathological confirmation. Importantly, the absence of a definitive imaging biomarker often leaves radiologists reliant solely on visual interpretation of breast DCE‐MRI. This limitation is crucial and should be carefully considered in discussions comparing the diagnostic roles of radiology and pathology.

Another key limitation of this study is the relatively small sample size, especially in subgroup analyses, which could impact the robustness and generalizability of our conclusions. As such, these findings should be interpreted with caution. However, the primary aim was to provide preliminary insights into the relationship between pathological response and MRI‐based radiologic response to neoadjuvant chemotherapy in non‐luminal breast cancer, regardless of whether the tumors presented as mass or non‐mass enhancement. These findings are based on the experience of a single institution. To strengthen the evidence base, future multicenter studies with larger and more diverse patient cohorts are warranted to validate the obtained findings and further explore the observed associations.

In our view, a major strength of this study lies in its robust support for the reliability of MRI in monitoring breast lesions during neoadjuvant therapy, particularly in non‐luminal breast cancer, regardless of whether the lesions present as mass or non‐mass enhancement. This represents a notable contrast to previous studies, which have largely focused on the utility of MRI in non‐luminal cases with mass‐like morphology. Our findings, therefore, expand the potential role of MRI, extending its applicability beyond the scope previously established in the literature.

## 5 Conclusion

Our findings indicate that MRI demonstrated a sensitivity of 86.4%, a specificity of 96.8%, and an overall accuracy of 92.4% in detecting residual disease among non‐luminal breast cancer subtypes. As such, it remains the most precise imaging modality for assessing tumor response to NACT and informing surgical planning. This high diagnostic performance is further reinforced by the strong correlation observed between MRI‐based estimations of residual disease and pathological outcomes. Notably, all MRI evaluations were conducted by experienced breast radiologists, likely contributing to the high diagnostic accuracy and substantial interobserver agreement observed. Our findings support the role of preoperative MRI in informing surgical decision‐making, particularly in assessing the feasibility of breast‐conserving surgery. By providing detailed tumor characterization and extent, MRI can assist in personalized surgical planning and potentially improve clinical outcomes by lowering positive margin rates and the need for reoperations. Nevertheless, these findings should be interpreted cautiously given the limited sample size. Larger and more extensive studies are needed to validate these results and further establish MRI's role in this clinical context. Future clinical trials are also encouraged to optimize and standardize imaging protocols, supporting a more integrated approach to evaluating treatment response in breast cancer care.

## Author Contributions

**Wesal M. Eldehna:** writing – review and editing, methodology, data collection. **Fawzy Elbarbry:** writing – review and editing, writing – final draft, data analysis. **Ahmed Elaryan:** visualization, validation, writing – original draft. **Rafat Abu Shakra:** visualization, validation, writing – original draft. **Abdul Hameed Hassan:** data curation, data analysis. **Ola Mousa Abdelfattah Elnady:** writing – review and editing. **Elshaimaa Mohamed Mohamed:** visualization, validation, writing – original draft.

## Ethics Statement

This retrospective single‐institutional study was approved by the institutional review board (approval number: 2023–11‐234) of the International Medical Center (IMC) in Jeddah, Saudi Arabia that waived the written informed consent requirement.

## Conflicts of Interest

The authors declare no conflicts of interest.

## Data Availability

The data that support the findings of this study are available on request from the corresponding author. The data are not publicly available due to privacy or ethical restrictions.
